# From study design to executable code: automating target trial emulation with large language models

**DOI:** 10.1093/jamiaopen/ooag131

**Published:** 2026-07-09

**Authors:** Hanjae Kim, Minseong Kim, Seonji Kim, Seng Chan You

**Affiliations:** Department of Biomedical Systems Informatics, Yonsei University College of Medicine, Seoul, Republic of Korea; Yonsei Institute for Digital Health, Yonsei University, Seoul, Republic of Korea; Institute for AI and Social Innovation, Yonsei University, Seoul, Republic of Korea; Department of Biomedical Systems Informatics, Yonsei University College of Medicine, Seoul, Republic of Korea; Department of Biomedical Systems Informatics, Yonsei University College of Medicine, Seoul, Republic of Korea; Yonsei Institute for Digital Health, Yonsei University, Seoul, Republic of Korea; Department of Biomedical Systems Informatics, Yonsei University College of Medicine, Seoul, Republic of Korea; Yonsei Institute for Digital Health, Yonsei University, Seoul, Republic of Korea; Institute for AI and Social Innovation, Yonsei University, Seoul, Republic of Korea

**Keywords:** target trial emulation, large language models, OHDSI, human-in-the-loop AI, reproducibility of results

## Abstract

**Objective:**

Implementing target trial emulation (TTE) studies as standardized, reproducible analytic workflows is technically demanding. We developed Text-guided Health-study Estimation and Specification Engine Using Strategus (THESEUS), which uses large language models (LLMs) to translate free-text study descriptions into structured analytic specifications and Strategus R scripts within the Observational Health Data Sciences and Informatics (OHDSI) ecosystem.

**Materials and Methods:**

THESEUS executes 2 steps: an LLM maps study descriptions to a JavaScript Object Notation (JSON) schema, and validated specifications are converted into Strategus R scripts through rule-based logic. For standardization evaluation, we compared specifications generated by 8 LLMs using 15 OHDSI-based TTE studies and 15 non-OHDSI studies under primary-analysis and full-analyses settings.

**Results:**

Under the primary-analysis setting, overall standardization accuracy ranged from 0.93 to 0.97 across models in OHDSI studies and from 0.82 to 0.95 in non-OHDSI studies. Gemini-3.1-Pro achieved the highest overall accuracy in OHDSI studies, while Gemini-3.1-Pro and Gpt-5.5 jointly achieved the highest overall accuracy in non-OHDSI studies. Under the full-analyses setting, field-level sensitivity ranged from 0.83 to 0.97 in OHDSI studies, with 0.07–0.80 false positives (FPs) per study, and from 0.77 to 0.89 in non-OHDSI studies, with 0.53–1.20 FPs per study. Gpt-5.5 performed best at the field level. THESEUS was implemented as a web application and coding-agent tools.

**Discussion:**

Pairing a standardized data model with a structured analysis framework enables reliable LLM-assisted interpretation of study descriptions and deterministic workflow construction in observational research.

**Conclusion:**

THESEUS supports translation of natural language study descriptions into executable, shareable code in standardized observational research settings.

## Background

Comparative effectiveness research (CER) using observational data has become indispensable for generating real-world evidence on treatment effects, particularly when randomized controlled trials are infeasible due to ethical, logistical, or financial constraints. Target trial emulation (TTE), a methodological framework that explicitly mirrors the protocol of a hypothetical randomized trial to estimate causal effects from observational data,^1^^,^^2^ has emerged as the standard approach for rigorous CER. Conducting a TTE study involves defining key components such as eligibility criteria, study periods, follow-up period with time zero, confounding control strategy, and outcomes,^1^ each of which must be translated into executable code. This translation from a conceptual study design to a working analytic program remains a critical bottleneck, requiring both methodological expertise in causal inference and programming proficiency in statistical languages such as R or Python. Recent reviews have identified significant gaps in tool support for TTE, noting that no single tool currently supports all phases from design through analysis,^3^ and that the primary barrier to broader adoption remains a lack of technical expertise rather than methodological understanding.^4^

The programming burden in CER is not merely a matter of inconvenience; it poses a fundamental barrier to the scalability and reproducibility of observational research. Each research team typically writes custom analytic code tailored to their local data environment, making it difficult to verify, replicate, or extend findings across institutions. Even when study designs are conceptually identical, differences in implementation such as variable naming, data transformations or even package versions can lead to divergent results. Addressing this challenge requires not only reducing the coding effort but also ensuring that the generated code operates on a standardized substrate where the same analytic logic can be faithfully executed across sites.

The Observational Health Data Sciences and Informatics (OHDSI)^5^ community provides precisely such a substrate. OHDSI has built an international research network in which participating organizations convert their local data into the Observational Medical Outcomes Partnership Common Data Model (OMOP CDM), an open community data standard that harmonizes both the structure and the vocabulary of observational data across diverse sources.^6^ Beyond data standardization, OHDSI has developed a comprehensive ecosystem of standardized open-source analytic tools. The Health Analytics Data-to-Evidence Suite (HADES)^7^ provides a collection of R packages for observational research, and Strategus^8^ orchestrates these modules into fully reproducible workflows by generating a single JavaScript Object Notation (JSON) specification that encapsulates the entire analysis. A study coordinator can share this JSON file with collaborators at any OMOP CDM-converted site, who can then execute the analysis without writing or modifying any code. This combination of a standardized data model with a standardized analysis framework creates a uniquely favorable environment for automating the code generation process. Once a study design is formally specified, the mapping to executable code becomes deterministic rather than ad hoc. However, Strategus remains fundamentally an R package, and both HADES and Strategus require familiarity with OHDSI-specific conventions, which continues to limit accessibility for researchers who lack coding experience or come from other methodological traditions.

Recent advances in large language models (LLMs) have demonstrated their potential to bridge the gap between natural language and structured computations across various healthcare domains, including clinical trial design,^9^ eligibility criteria conversion,^10^^,^^11^ and electronic health record phenotyping.^12^ We hypothesized that the standardized nature of OMOP CDM, combined with the well-defined structure of Strategus analysis specifications, would make this ecosystem particularly amenable to LLM-assisted standardization because the target representation is constrained and predictable rather than open ended. Inspired by prior work on SOCRATex,^13^ a hierarchical standardization framework that transforms free-text clinical narratives into structured, machine-readable representations, we developed a 2-step process consisting of LLM-assisted standardization and deterministic workflow generation. The developed framework, Text-guided Health-study Estimation and Specification Engine Using Strategus (THESEUS), standardizes natural language study descriptions into structured JSON-based analytic specifications and then converts these specifications into executable Strategus R scripts through rule-based logic. THESEUS aims to make TTE more accessible to a broader range of researchers by lowering the learning curve for those entering the OHDSI ecosystem, and to enhance the reproducibility of observational studies conducted across the OHDSI network.

## Methods

We report the details of this study in accordance with the Minimum Reporting Items for Clear Evaluation of Accuracy Reports of Large Language Models in Healthcare (MI-CLEAR-LLM) checklist.^14^

### Conceptual framework design

**Figure 1.**
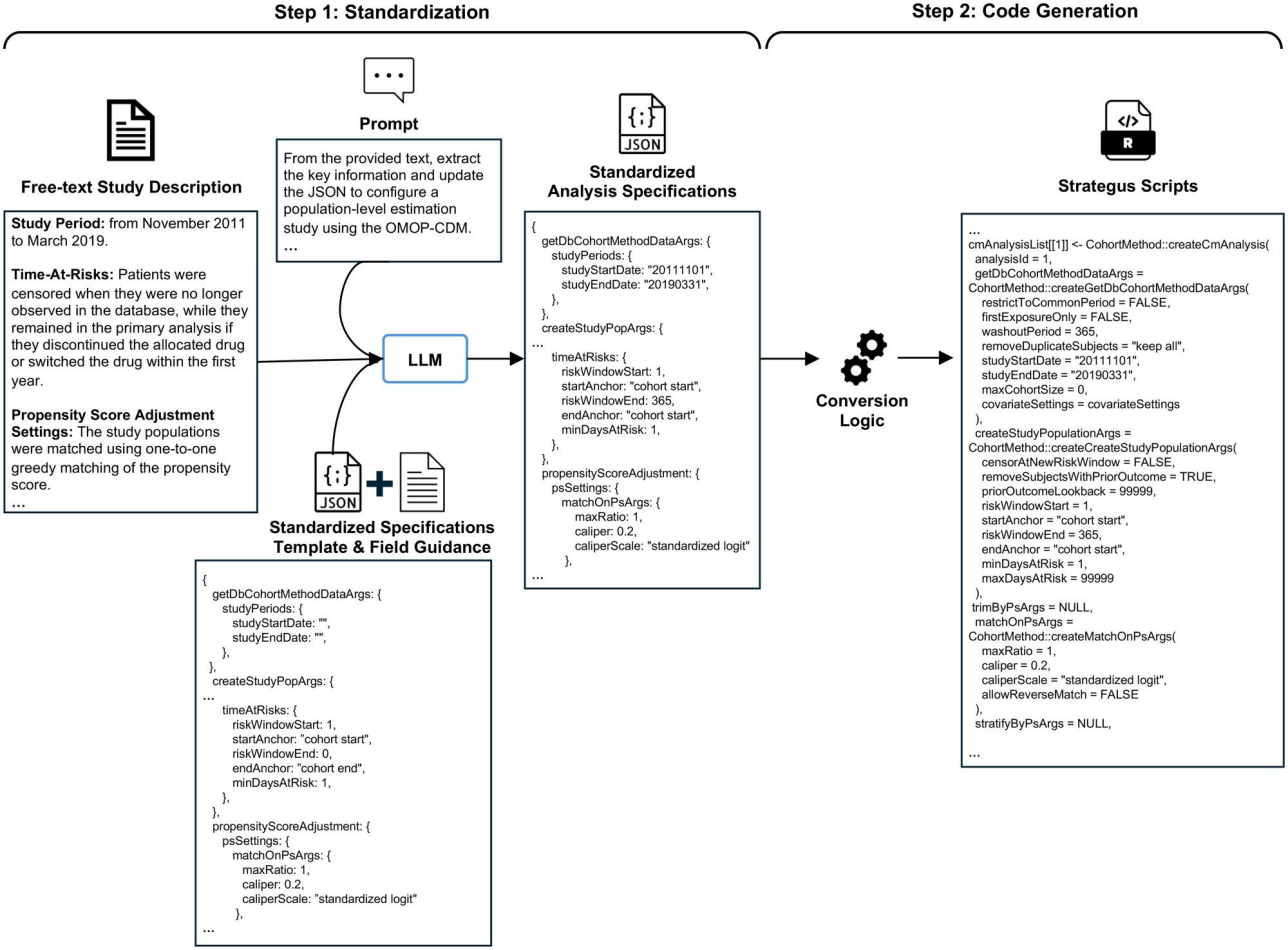
Conceptual framework of THESEUS for translating free-text study descriptions into Strategus scripts. LLM, large language model.

As proof-of-concept, we focused on implementing the “Cohort Method”^15^ design within the OHDSI research framework which emulates randomized controlled trials. This requires specifying the study period, time-at-risk (TAR) defined as follow-up window, propensity score (PS) adjustment strategy for confounding control, and outcome model settings. We adopted a 2-step approach consisting of standardization and code generation to translate natural language study descriptions into Strategus-compatible analytic code (Figure 1).

### Step 1: Standardizing free-text study descriptions into structured specifications

When a researcher plans a study in natural language, this needs to be translated into a standardized OHDSI research framework. We leveraged a JSON-based schema that is commonly used within the OHDSI ecosystem to represent study design components. This schema encompasses the core design components required to specify an observational study. In this study, we restricted its scope to 4 core fields relevant to Cohort Method implementation: study period, TAR, PS adjustment, and outcome model (Supplementary Material 1). The schema allowed multiple entries within each field group so that primary and sensitivity analyses could be represented within a single structured specification. An LLM receives text descriptions of study designs, the schema template, and field-level guidance (Supplementary Material 2) derived from the OHDSI guideline book^5^ as inputs and generates a structured specification in JSON format. In addition, the LLM produces an explanation describing how each part of the text was interpreted and applied to the analytic specifications. The prompt used for this standardization step is provided in Supplementary Material 3.

### Step 2: Generating Strategus R scripts based on structured study specifications

The structured specifications generated in the previous step are then transformed into executable Strategus R scripts. This conversion is performed through deterministic rule-based logic, which was feasible because HADES modules are standardized and Strategus scripts follow recurring implementation patterns for analyses. The resulting scripts are provided with an accompanying “renv”^16^ environment that records and restores the required R package environment, including the HADES packages used by the generated Strategus scripts (details of package versions used in this study are reported in Supplementary Material 4). This allows users to directly execute the scripts without modification. Upon execution, the script generates the final JSON file required to run the actual analysis, which can then be shared directly with collaborating researchers.

### Prototype implementation and workflow integration

**Figure 2.**
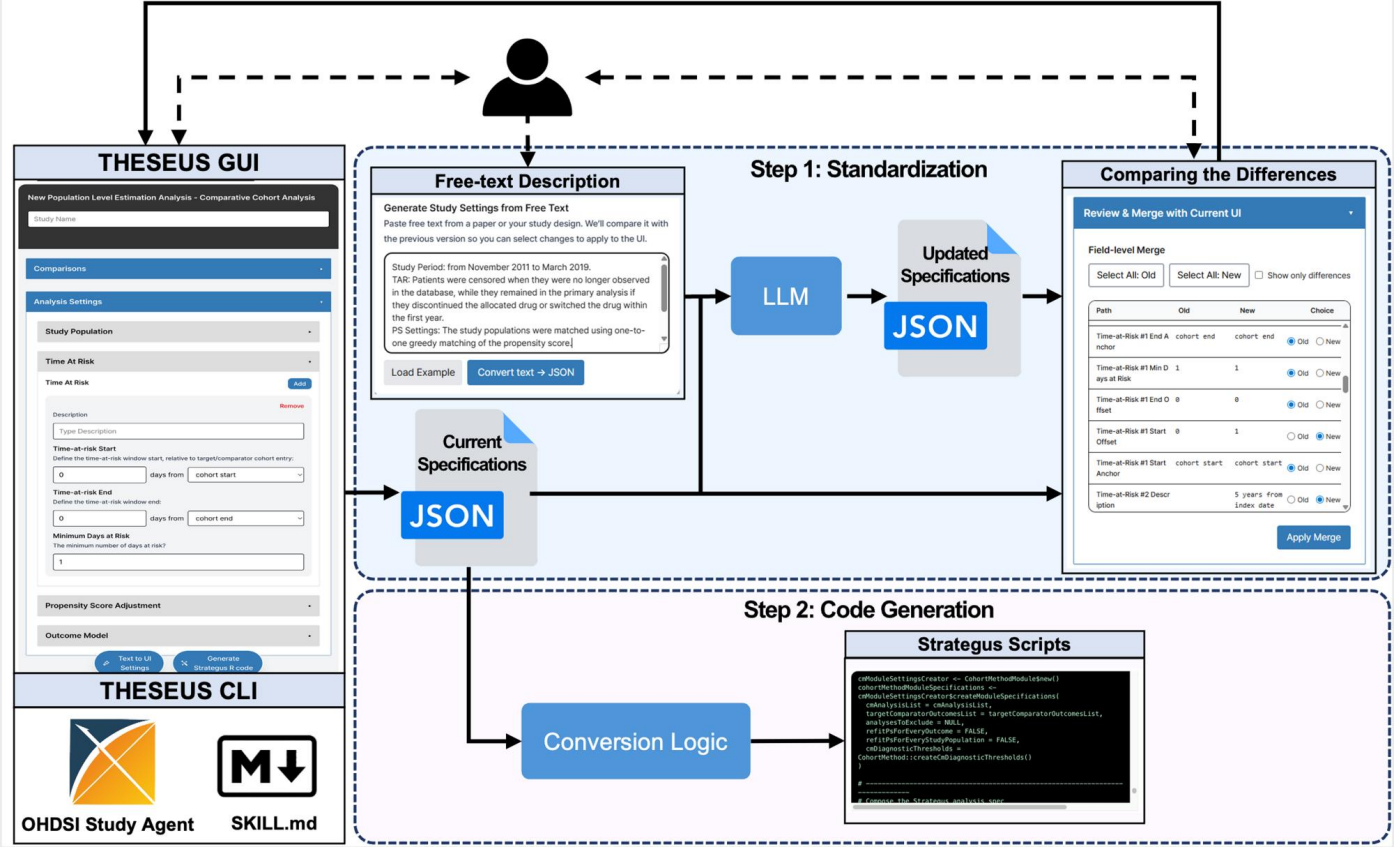
Overall workflow of THESEUS powered by LLMs with human-in-the-loop configuration. The dashed arrows represent the interaction between human and the system. GUI, graphical user interface; LLM, large language model; JSON, JavaScript Object Notation.

The OHDSI community provides a web-based platform called ATLAS (https://atlas-demo.ohdsi.org/), which provides an environment for configuring various analysis specifications within a unified graphical user interface (GUI). To enable human-in-the-loop validation of the standardized specifications before conducting the code generation step, we developed a GUI prototype that resembles the “population-level estimation” tab of ATLAS version 2.14.1 (Supplementary Material 5), incorporating the proposed agentic framework. In the GUI workflow, users can configure analysis settings with manual clicks, while they can also input free-text study descriptions, which will be translated into corresponding analysis specifications displayed on the GUI. Before updating the configuration, the system presents a side-by-side comparison of the original and revised specifications, allowing users to selectively accept or reject individual modifications. Once the configuration is finalized, a single click of a button enables conversion of the GUI-based analysis specifications into executable Strategus R scripts. Figure 2 illustrates the overall workflow. To support additional research workflows, the same standardization and conversion functions were also implemented as command-line interface (CLI) accessible tooling that can be integrated into diverse research workflows.

### Evaluation of the framework

Previously published TTE study papers^17–31^ that used OMOP CDM and OHDSI R packages (either HADES or Strategus) were used as natural language samples to evaluate the framework’s ability to standardize free-text study descriptions into structured analytic specifications. To assess the generalizability of the framework beyond the OHDSI community, we additionally used non-OHDSI TTE study papers.^32–46^ We included non-OHDSI studies that explicitly described the follow-up window, PS adjustment, and outcome model settings. Studies whose core analyses required settings outside OHDSI’s supported Cohort Method scope were excluded; for example, we excluded studies that used outcome models other than Cox, Poisson, or logistic regression. All texts describing study period, TAR, PS adjustment, and outcome model settings were manually extracted by the authors. For both OHDSI and non-OHDSI studies, we evaluated 2 input settings: (1) primary-analysis text only and (2) full-analyses text including primary and sensitivity analyses. The full-analyses setting allowed the generated JSON specifications to include multiple fields within each section of the schema (e.g., multiple risk windows within the TAR section for a single study). Across both input settings, each study was processed using independent LLM inferences, and the resulting structured specifications were compared with gold standards created by the authors. For the primary-analysis setting, accuracy was assessed separately for each of the 4 specification sections at the study level. For the full-analyses setting, where a single section could contain multiple fields, we performed field-level evaluation, treating each subfield as an independent unit and aggregating them across all studies to compute sensitivity, precision, and false positives (FPs) per study.

For OHDSI studies only, we further tested augmented descriptions of the primary settings. For each study, 2 augmented versions were generated from the primary-analysis description by preserving the original text structure and wording as much as possible while recombining the study design differently. This augmented-primary setting was used to assess model robustness to modified study-design scenarios. All augmented-primary texts are publicly available at https://github.com/dr-you-group/theseus-evaluation/tree/main/public/goldStandard/primary_augmented.

For all evaluations, we compared a diverse set of proprietary LLMs from 4 major vendors: OpenAI Gpt-5.5, Gpt-5.4-mini), Google (Gemini-3.1-Pro, Gemini-3.1-Flash-Lite), Anthropic (Claude-opus-4.8, Claude-haiku-4.5), and DeepSeek (Deepseek-v4-Pro, Deepseek-v4-Flash). Detailed model versions are specified in Supplementary Material 6. All models were accessed via their application programming interfaces (APIs). No model fine-tuning was performed in this study. All model queries were executed in June 2026. To minimize variability across inference attempts, the temperature parameter was set to 0. Each input prompt was executed as a single attempt without retries. Across all vendors, we passed the same prompt content, without any system message or vendor-specific modification.

## Results

### Resulting prototypes and public resources

The current prototype is available as both a web-based GUI (https://theseus2.vercel.app/) and CLI-oriented workflows (https://github.com/dr-you-group/theseus-plugin.git). In the CLI workflow, AI coding agents can access THESEUS functionality as Model Context Protocol tools and slash commands, supporting study description standardization, specification validation, and generation of Strategus code. In parallel, we have also incorporated the framework into OHDSI’s ongoing Study Agent initiative (https://github.com/OHDSI/StudyAgent), an agent-style study design assistant that supports end-to-end OHDSI study workflows including Cohort Method analysis.

### Standardization evaluation results

Under the primary-analysis setting, across the 15 OHDSI-based studies, overall accuracy ranged from 0.93 to 0.97 across models, with Gemini-3.1-Pro achieving the highest overall accuracy (study period 1.00, TAR 0.93, PS adjustment 0.93, outcome model 1.00) (Table 1). Across the 15 non-OHDSI studies, overall accuracy under the primary-analysis setting ranged from 0.82 to 0.95, with Gpt-5.5 and Gemini-3.1-Pro achieving the highest (Gpt-5.5: study period 0.93, TAR 0.87, PS adjustment 1.00, outcome model 1.00; Gemini-3.1-pro: study period 0.93, TAR 0.93, PS adjustment 0.93, outcome model 1.00).

**Table 1.** Study-level standardization accuracy across OHDSI and non-OHDSI studies under primary-analysis input setting.

Models	OHDSI studies (*N* = 15)					Non-OHDSI studies (*N* = 15)				
	Accuracy									
	SP	TAR	PS	OM	Overall	SP	TAR	PS	OM	Overall
Gpt-5.5	**1.00**	0.87	0.93	**1.00**	0.95	**0.93**	0.87	**1.00**	**1.00**	**0.95**
Gpt-5.4-mini	**1.00**	0.80	**1.00**	**1.00**	0.95	0.87	0.53	0.93	**1.00**	0.83
Gemini-3.1-Pro	**1.00**	**0.93**	0.93	**1.00**	**0.97**	**0.93**	**0.93**	0.93	**1.00**	**0.95**
Gemini-3.1-Flash-Lite	**1.00**	0.80	**1.00**	**1.00**	0.95	0.87	0.53	**1.00**	0.87	0.82
Claude-opus-4.8	**1.00**	0.73	**1.00**	**1.00**	0.93	0.87	0.73	0.93	0.93	0.87
Claude-haiku-4.5	**1.00**	0.80	**1.00**	**1.00**	0.95	0.87	0.60	**1.00**	**1.00**	0.87
DeepSeek-V4-Pro	**1.00**	0.73	**1.00**	**1.00**	0.93	**0.93**	**0.93**	0.93	0.93	0.93
DeepSeek-V4-Flash	**1.00**	0.80	**1.00**	**1.00**	0.95	**0.93**	0.67	0.93	0.93	0.87

Bold values indicate the highest score(s) within each column. OHDSI, Observational Health Data Sciences and Informatics; OM, outcome model; PS, propensity score (adjustment); SP, study period; TAR, time-at-risk.

At the field-level evaluation for the full-analyses setting, the 15 OHDSI studies yielded 99 gold standard specification items (study period 18, TAR 35, PS adjustment, 31, outcome model 15). Precision ranged from 0.87 to 0.99, sensitivity from 0.83 to 0.97, with FPs per study ranging from 0.07 to 0.80 across models (Table 2). Gpt-5.5 achieved the strongest field-level performance (precision 0.99, sensitivity 0.97, FPs per study 0.07). For the 15 non-OHDSI studies, 83 gold standard specification items were evaluated under the full-analyses setting (study period 22, TAR 20, PS adjustment 22, outcome model 19). Precision ranged from 0.78 to 0.90, sensitivity from 0.77 to 0.89, with FPs per study ranging from 0.53 to 1.20. Gpt-5.5 achieved the strongest field-level performance (precision 0.90, sensitivity 0.89, FPs per study 0.53).

**Table 2.** Field-level performance across OHDSI and non-OHDSI studies under full-analyses input setting.

Models	OHDSI studies (*n* = 99)			Non-OHDSI studies (*n* = 83)		
	Precision	Sensitivity	FPs per study	Precision	Sensitivity	FPs per study
Gpt-5.5	**0.99**	**0.97**	**0.07**	**0.90**	**0.89**	**0.53**
Gpt-5.4-mini	0.87	0.83	0.80	0.87	0.86	0.73
Gemini-3.1-Pro	0.96	0.93	0.27	0.89	0.88	0.60
Gemini-3.1-Flash-lite	0.94	0.90	0.40	0.78	0.77	1.20
Claude-opus-4.8	0.97	0.95	0.20	0.88	0.86	0.67
Claude-haiku-4.5	0.95	0.92	0.33	0.83	0.82	0.93
DeepSeek-V4-Pro	0.95	0.93	0.33	0.89	0.87	0.60
DeepSeek-V4-Flash	**0.99**	0.94	**0.07**	0.84	0.83	0.87

Bold values indicate the highest score(s) within each column. OHDSI, Observational Health Data Sciences and Informatics; FPs, false positives.

**Figure 3.**
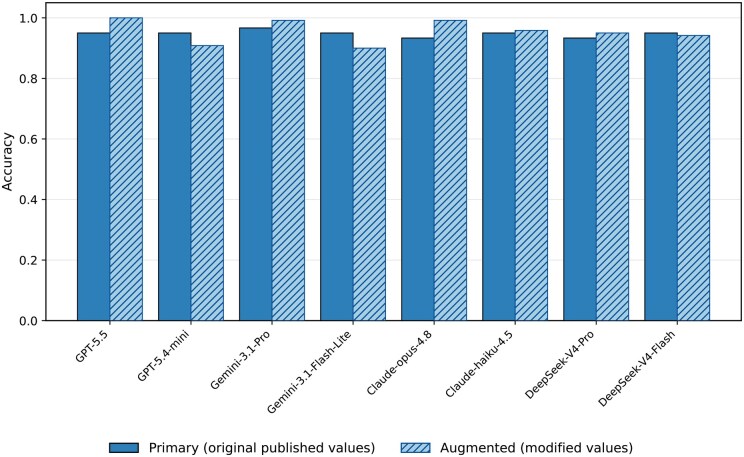
Overall accuracy of LLMs on primary and augmented-primary OHDSI study protocols. The solid bars represent the primary-analysis input condition and the hatched bars represent the augmented-primary condition. Overall accuracy is the mean of the four analytic specification sections: study period, time-at-risk, propensity score adjustment, and outcome model. ; LLM, large language model; OHDSI, Observational Health Data Sciences and Informatics.

In the augmented-primary setting for OHDSI studies, 2 augmented texts were generated per study, yielding 30 augmented descriptions. Overall accuracy ranged from 0.82 to 1.00 (Figure 3), with Gpt-5.5 achieving perfect accuracy across all 4 specification sections (Supplementary Material 7).

## Discussion

This study demonstrates that implementation of study designs as TTE workflows can be substantially automated when the underlying data and analytic infrastructure are standardized. By leveraging the structured nature of OMOP CDM and the well-defined specification format of Strategus, THESEUS uses LLMs to translate natural language study descriptions into structured, machine-readable analytic specifications that can be reviewed by humans and then converted into scripts for executable workflows. Our results show that current LLMs can extract core analytic parameters from natural language study descriptions with high accuracy, suggesting that LLMs can support research standardization by transforming heterogeneous textual descriptions into a common specification format. Once researchers review and finalize the structured specification, this specification can be deterministically converted into Strategus scripts that follow standardized, error-free implementation patterns, providing a reliable end-to-end path from protocol interpretation to executable analysis workflows.

A key insight from this work is that the success of this automated workflow critically depends on the degree of standardization in both the data layer and the analytic layer. Even when observational studies address the same research question, independent research teams may implement the study design differently, leading to heterogeneous results that reduce the reproducibility of the studies.^47^,^48^ OHDSI’s ecosystem resolves this problem through 2 layers of standardization: OMOP CDM provides a universal data structure and vocabulary, while Strategus provides a universal specification format for analyses. Together, these layers reduce the workflow-generation task from an open-ended programming problem to a structured mapping problem, where the input (a JSON specification) and the output (an R script) both conform to well-defined schemas. This is what makes the OHDSI ecosystem uniquely amenable to automation, and we believe other standardized research platforms in diverse domains could similarly benefit from LLM-assisted study workflow generation.

Our evaluation of the standardization step showed that LLMs have strong potential to extract core analytic settings from natural‑language descriptions, though performance varied across specification components. Study period, PS adjustment, and outcome model specifications were extracted with consistently high accuracy across models, whereas TAR extraction remained more challenging, likely reflecting the greater ambiguity and diversity in how this parameter is described in published methods sections. When sensitivity analyses were included, overall field-level performance showed high sensitivity with low false positive rates, with the best-performing models achieving high precision and sensitivity in both OHDSI and non-OHDSI studies while maintaining fewer than one false positive per study. The external validation with non-OHDSI studies further demonstrates that the standardization step can generalize beyond the OHDSI community, enabling retrospective reconstruction of analytic specifications from studies conducted in different data environments.

Cohort definition remains one of the most complex and labor-intensive steps in observational studies,^49^ and complementary efforts are advancing its automation. Park et al. developed Criteria2Query 3.0,^11^ a system that transforms free-text clinical trial eligibility criteria into executable OMOP CDM–compatible queries using LLMs for concept extraction and structured query language generation. More recently, Lee et al. proposed a similar framework with systematic evaluation across multiple LLMs.^10^ Integrating such cohort definition pipelines with THESEUS could enable end-to-end automation of the entire TTE workflow, from defining study populations to generating executable analysis code. Furthermore, as demonstrated by our external validation, such an integrated system could also facilitate retrospective verification of previously published studies by reconstructing their analytic specifications from reported methods.

This study has several limitations. First, the current prototype is limited to the TTE framework. However, the modular architecture of THESEUS can be expanded to support a broader range of study designs such as characterization or patient-level prediction. Second, the present evaluation focused on 4 core specification components: study period, TAR, PS adjustment, and outcome model, and other detailed settings relevant to TTE implementation were not evaluated. Third, the current system covers only the subset of TTE settings that can be represented within the OHDSI Cohort Method workflow. Accordingly, study papers that relied on unsupported analytical choices were excluded from evaluation; examples include studies using outcome models other than Cox, Poisson, or logistic regression, as well as doubly robust estimation. Fourth, we were unable to conduct a large-scale evaluation across the full diversity of published TTE studies, because constructing gold standard specifications from published papers is labor-intensive and requires domain expertise. Fifth, although our augmented-primary analysis suggests that model performance reflects genuine extraction rather than memorization, we cannot fully rule out the possibility that exposure to the source publications during pretraining contributed to the observed accuracy, because the composition of these models’ training corpora is not publicly available. Sixth, our evaluation focused only on the accuracy of LLM-generated specifications and did not assess the usability among actual researchers. Direct evaluation is needed to determine whether this framework reduces time-to-completion, cognitive load of researchers, or the learning curve for conducting OHDSI studies. We are extending this framework into a comprehensive AI agent for designing OHDSI studies, and future work will evaluate its practical usability in real research settings. Finally, the framework’s reliance on OHDSI-specific tools means that researchers at institutions without OMOP CDM-converted databases would need to undergo data conversion before benefiting from automated code generation—though the growing global adoption of OMOP CDM is progressively lowering this barrier.

In conclusion, THESEUS demonstrates that the combination of standardized data models and analysis frameworks creates a fertile ground for LLM-assisted generation of study workflows in CER. This work addresses a key bottleneck that has limited the scalability and accessibility of observational studies by enabling researchers to express study designs in natural language. As standardized data networks continue to expand globally, the principles of leveraging structural constraints to enable reliable automation may generalize to other research ecosystems and study types, ultimately broadening participation in evidence generation from real-world data.

## Supplementary Material

ooag131_Supplementary_Data

## Data Availability

The source code for the web-based application is available at https://github.com/dr-you-group/theseus-app. The CLI-oriented tooling is available at https://github.com/dr-you-group/theseus-plugin.git. The source code and data needed for the evaluation are available at https://github.com/dr-you-group/theseus-evaluation. The data are also available in the Dryad Digital Repository at https://doi.org/10.5061/dryad.qnk98sfzs.
