# Supplementary material for: From study design to executable code: automating target trial emulation with large language models
Source: JAMIA Open. 2026 Jul 9;9(4):ooag131. doi: 10.1093/jamiaopen/ooag131 (PMC13348706; doi:10.1093/jamiaopen/ooag131)

**Supplementary Material 1.** The predefined JSON template for the analysis specifications in the standardization step

| {  "getDbCohortMethodDataArgs": {  "studyPeriods": [  {  "description": "",  "studyStartDate": "",  "studyEndDate": ""  }  ],  "firstExposureOnly": false,  "removeDuplicateSubjects": "keep all",  "restrictToCommonPeriod": false,  "washoutPeriod": 365,  "maxCohortSize": 0  },  "createStudyPopArgs": {  "removeSubjectsWithPriorOutcome": true,  "priorOutcomeLookback": 99999,  "timeAtRisks": [  {  "description": "",  "minDaysAtRisk": 1,  "riskWindowStart": 1,  "startAnchor": "cohort start",  "riskWindowEnd": 0,  "endAnchor": "cohort end"  }  ],  "censorAtNewRiskWindow": false  },  "psSettings": [  {  "description": "",  "trimByPsArgs": null,  "matchOnPsArgs": {  "maxRatio": 1,  "caliper": 0.2,  "caliperScale": "standardized logit"  },  "stratifyByPsArgs": null,  "inversePtWeighting": false  }  ],  "createPsArgs": {  "maxCohortSizeForFitting": 250000,  "errorOnHighCorrelation": true,  "prior": {  "priorType": "laplace",  "useCrossValidation": true  },  "control": {  "tolerance": 2e-7,  "cvType": "auto",  "fold": 10,  "cvRepetitions": 10,  "noiseLevel": "silent",  "resetCoefficients": true,  "startingVariance": 0.01  }  },  "fitOutcomeModelArgs": {  "outcomeModels": [  {  "description": "",  "modelType": "cox",  "useCovariates": false  }  ],  "stratified": false,  "prior": {  "priorType": "laplace",  "useCrossValidation": true  },  "control": {  "tolerance": 2e-7,  "cvType": "auto",  "fold": 10,  "cvRepetitions": 10,  "noiseLevel": "quiet",  "resetCoefficients": true,  "startingVariance": 0.01  }  }  } |
| --- |

**Supplementary Material 2.** Field-Level Guidance and Generation Rules for CohortMethod Analysis Specifications

| # CohortMethod cmAnalysis Field Notes  ### `getDbCohortMethodDataArgs`  - `studyStartDate`, `studyEndDate`: date strings in `yyyyMMdd` format, or blank  strings when not restricted.  - Meaning: Study start and end dates can be used to limit the analyses to a  specific period. The study end date also truncates risk windows, meaning no  outcomes beyond the study end date will be considered. Leave blank to use  all time.  - `firstExposureOnly`: `true` or `false`.  - Meaning: Can be used to restrict to the first exposure per patient.  - `removeDuplicateSubjects`: one of `keep all`, `keep first`, `remove all`,  or `keep first, truncate to second`.  - Meaning: What happens when a subject is in both target and comparator  cohorts. `keep all` keeps subjects in both cohorts, which can double-count  subjects and outcomes. `keep first` keeps the subject in the first cohort  that occurred. `remove all` removes the subject from both cohorts.  `keep first, truncate to second`.  - `restrictToCommonPeriod`: `true` or `false`.  - Meaning: Should the study be restricted to the period when both exposures  are present, such as when both drugs are on the market?  - `washoutPeriod`: non-negative integer number of days.  - Meaning: The minimum required continuous observation time prior to index  date for a person to be included in the cohort.  - `maxCohortSize`: non-negative integer; `0` means no maximum.  - Meaning: If either the target or the comparator cohort is larger than this number it will be sampled to this size.  ### `createStudyPopArgs`  - `removeSubjectsWithPriorOutcome`: `true` or `false`.  - Meaning: We can choose to remove subjects that have the outcome prior to  the risk window start.  - `priorOutcomeLookback`: non-negative integer lookback window.  - Meaning: If we choose to remove people who had the outcome before, this  controls how many days to look back when identifying prior outcomes.  - `minDaysAtRisk`: non-negative integer.  - Meaning: A patient with zero days at risk adds no information, so the minimum days at risk is normally set at one day. If there is a known latency for the side effect, then this may be increased to get a more informative proportion.  - `riskWindowStart`, `riskWindowEnd`: integer offsets from the selected anchor.  - Meaning: Time-at-risk can start one day after cohort start, so one day  after treatment initiation. Starting later than cohort start can exclude  outcome events on the day of treatment initiation when they are not  considered biologically plausible consequences of the drug. Time-at-risk  can end at cohort end, meaning when exposure stops, for an on-treatment  design. It can also end at a fixed duration after cohort entry regardless  of whether exposure continues, for an intent-to-treat design. A very large  end offset, such as 99999 days after cohort entry, effectively follows  subjects until observation end.  - `startAnchor`, `endAnchor`: one of `cohort start` or `cohort end`.  - Meaning: The anchors determine whether the risk-window offsets are measured  from cohort start or cohort end.  - `censorAtNewRiskWindow`: `true` or `false`.  - Meaning: If duplicate-subject options such as `keep all` or `keep first`  are selected, we may wish to censor the time when a person is in both  cohorts.  ### `trimByPsArgs`  - (default) Use `null` when no PS trimming is selected.  - Meaning: We can opt to trim the study population, removing people with extreme PS values. We can choose to remove the top and bottom percentage, or we can remove subjects whose preference score falls outside the range we specify. Trimming the cohorts is generally not recommended because it requires discarding observations, which reduces statistical power. It may be desirable to trim in some cases, for example when using IPTW.  - `trimFraction`: For percent trimming (a fraction, so 5 percent is represented as `0.05`). Default is `0.05`. Set `null` if equipose trimming.  - `equipoiseBounds`: For equipoise trimming. Default is `[25, 75]` Set `null` if percent trimming.  ### `matchOnPsArgs`  - Use an object only when matching on propensity score. Use `null` when stratifying by PS or when no PS adjustment is selected.  - Meaning: We can choose to match on the propensity score. When matching,  specify the maximum number of people from the comparator group to match to  each person in the target group, and specify the caliper.  - `maxRatio`: a non-negative integer; `0` means no maximum. Default is `1`.  - Meaning: The maximum number of people from the comparator group to match to  each person in the target group. Typical values are `1` for one-to-one  matching or a large number, such as `100`, for variable-ratio matching.  - `caliper`: numeric; `0` means no caliper is used. Default is `0.2`.  - Meaning: The maximum allowed difference between propensity scores to allow  a match.  - `caliperScale`: one of `propensity score`, `standardized`, or `standardized logit`. Default is `standardized logit`.  - Meaning: The caliper can be defined on the propensity score scale, the  standardized scale in standard deviations of the propensity score  distributions, or the standardized logit scale in standard deviations after  logit transformation to make the propensity score more normally  distributed.  ### `stratifyByPsArgs`  - Use an object only when stratifying by propensity score. Use `null` when matching on PS or when no PS adjustment is selected.  - Meaning: We can choose to stratify on the propensity score.  - `numberOfStrata`: a positive integer. Default is `5`.  - Meaning: When stratifying, specify the number of strata.  - `baseSelection`: one of `all`, `target`, or `comparator`. Default is `all`.  - Meaning: When stratifying, specify whether strata are based on the target,  comparator, or entire study population.  ### `inversePtWeighting`  - `true` or `false`.  - Meaning: Instead of stratifying or matching on the propensity score, inverse  probability of treatment weighting can be used.  ### `createPsArgs`  - Use `null` when no PS model is needed.  - `maxCohortSizeForFitting`: a non-negative integer; `0` means no downsampling.  - Meaning: The maximum number of people to include in the propensity score  model when fitting.  - `errorOnHighCorrelation`: `true` or `false`.  - Meaning: If any covariate has an unusually high correlation, either  positive or negative, this will throw an error.  - `prior` and `control` are `null` when regularization is disabled.  - `prior.priorType`: currently `laplace`.  - Meaning: Specify the prior distribution.  - `prior.useCrossValidation`: `true` or `false`.  - Meaning: Perform cross-validation to determine prior variance.  - `control.cvType`: `auto` or `grid`.  - Meaning: Cross-validation search type.  - `control.noiseLevel`: `silent`, `quiet`, or `noisy`.  - Meaning: Noise level for Cyclops screen output.  - `control.startingVariance`: numeric; `-1` means estimate from data.  - Meaning: Starting variance for auto-search cross-validation. `-1` means use  an estimate based on the data.  - `control.tolerance`: numeric convergence tolerance.  - Meaning: Maximum relative change in convergence criterion from successive  iterations.  - `control.fold`: number of random folds.  - Meaning: Number of random folds to employ in cross-validation.  - `control.cvRepetitions`: number of cross-validation repetitions.  - Meaning: Number of repetitions of cross-validation.  - `control.resetCoefficients`: `true` or `false`.  - Meaning: Reset all coefficients to 0 between model fits under  cross-validation.  ### `fitOutcomeModelArgs`  - `modelType`: one of `logistic`, `poisson`, or `cox`.  - Meaning: The statistical model used to estimate the relative risk of the  outcome between target and comparator cohorts.  - `stratified`: `true` or `false`.  - Meaning: Whether the regression should be conditioned on the strata. For  one-to-one matching this is likely unnecessary and may lose power. For  stratification or variable-ratio matching it is required.  - `useCovariates`: `true` or `false`.  - Meaning: Covariates can be added to the outcome model to adjust the  analysis. The recommended default is to keep the outcome model as simple as  possible and not include additional covariates.  - `prior` and `control` are `null` when regularization is disabled.  - `prior.priorType`: currently `laplace`.  - Meaning: Specify the prior distribution.  - `prior.useCrossValidation`: `true` or `false`.  - Meaning: Perform cross-validation to determine prior variance.  - `control` follows the same field conventions as `createPsArgs.control`.  - Meaning: The outcome-model control object uses the same conventions for  tolerance, cross-validation type, folds, repetitions, noise level,  coefficient reset, and starting variance as `createPsArgs.control`.  ## Generation Rules  - Matching and stratification are mutually exclusive:  - `matchOnPsArgs` object and `stratifyByPsArgs = null`, or  - `matchOnPsArgs = null` and `stratifyByPsArgs` object, or  - both `null` when no PS adjustment is selected.  - If trimming is selected without matching or stratification, `createPsArgs`  should still be present because PS values are required for trimming.  - The generated artifact should be valid JSON with no comments. |
| --- |

**Supplementary Material 3.** Prompt used for standardization step in THESEUS

${text} denotes the user’s study design described in natural language, ${analysisSpecificationsTemplate} refers to the predefined JSON template that constrains the output structure of the generated analysis specifications, and ${jsonFieldDescriptions} refers to the accompanying field descriptions for the template. The content of ${jsonFieldDescriptions} is directly taken from the field descriptions provided in Supplementary 2.

| <Instruction>  From the provided <Text>, extract the key information to configure a population-level estimation study using the OMOP-CDM.  Leave any settings at their default values if they are not specified in the <Text>.  Refer to the fields and value types provided in the <Analysis Specifications Template> and do not add any additional fields.  For each field, refer to <JSON Fields Descriptions> to ensure accurate mapping of the relevant information from <Text> to the corresponding JSON structure.  Additional sensitivity analyses beyond the primary analysis may have also been conducted. If the text describes multiple settings for a field (e.g., more than one timeAtRisk window), produce a separate entry for each setting within its corresponding array.  </Instruction>    <Text>  ${text}  </Text>    <Analysis Specifications Template>  ${analysisSpecificationsTemplate}  </Analysis Specifications Template>  <JSON Fields Descriptions>  {jsonFieldDescriptions}  </JSON Fields Descriptions> |
| --- |

**Supplementary Material 5.** User interfaces of the original ATLAS (left) and THESEUS (right)

| 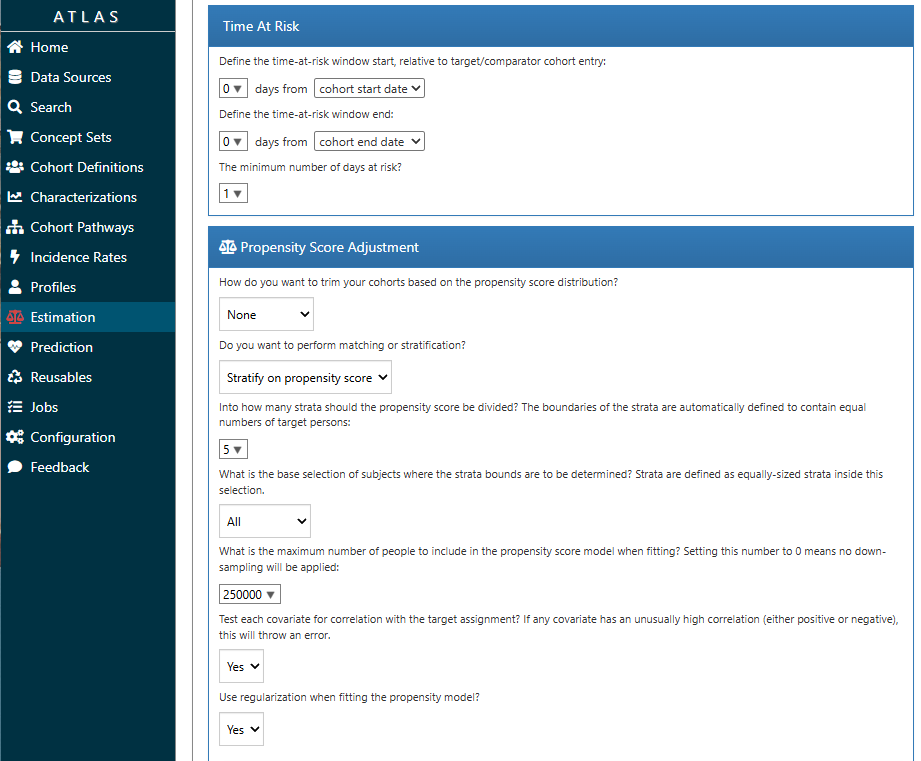 | 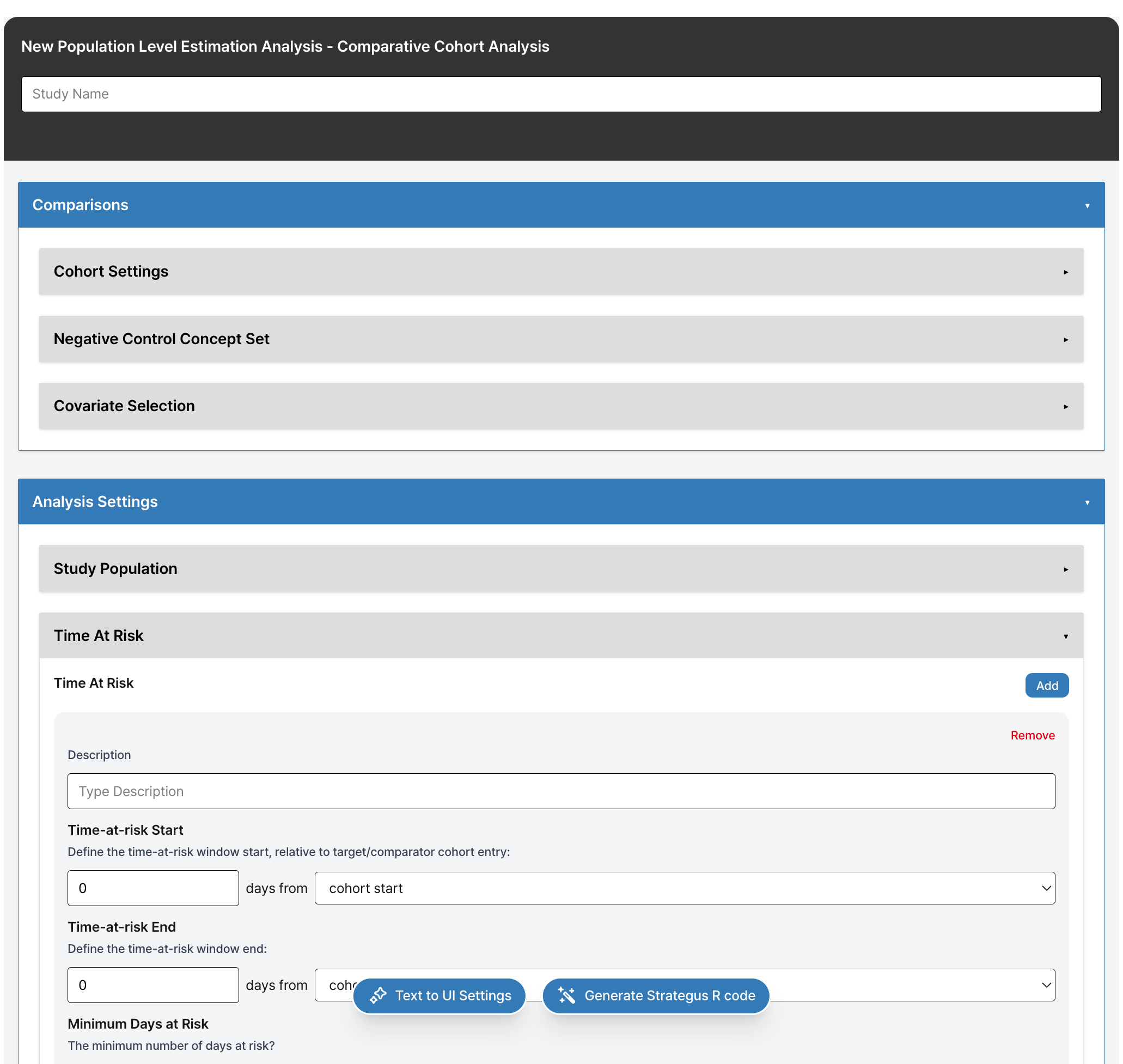 |
| --- | --- |

**Supplementary Material 6.** Large Language Model specifications

| **Vendor** | **Model** | **Model release date** | **knowledge cutoff** |
| --- | --- | --- | --- |
| OpenAI | GPT-5.5 | 2026-04-23 | 2025-12 |
|  | GPT-5.4-mini | 2026-03-17 | 2025-08 |
| Gemini | Gemini-3.1-Pro (Preview) | 2026-02-19 | 2025-01 |
|  | Gemini-3.1-Flash-Lite | 2026-03-03 | 2025-01 |
| Claude | Claude-Opus-4.8 | 2026-05-28 | 2026-01 |
|  | Claude-Haiku-4.5 | 2025-10-01 | 2025-02 |
| Deepseek | DeepSeek-V4-Pro | 2026-04-24 | unknown |
|  | DeepSeek-V4-Flash | 2026-04-24 | unknown |

**Supplementary Material 7.** Per-section accuracy across primary and value-augmented protocols


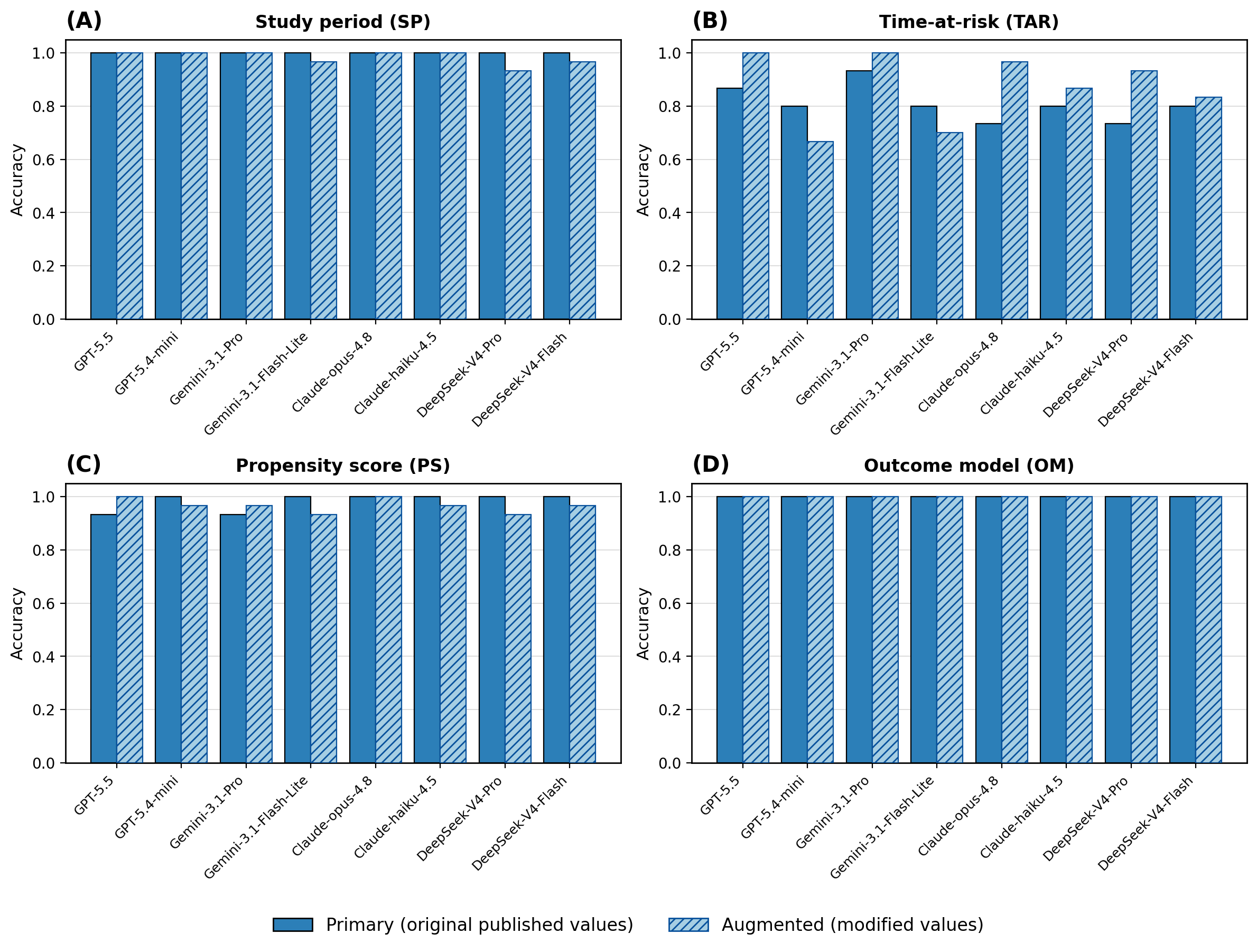

Supplement: ooag131_Supplementary_Data [file ooag131_supplementary_data.zip › Supplementary Materials_revised.docx]
